# Identifying the paths of climate effects on population dynamics: dynamic and multilevel structural equation model around the annual cycle

**DOI:** 10.1007/s00442-020-04817-3

**Published:** 2021-01-18

**Authors:** Vesa Selonen, Samuli Helle, Toni Laaksonen, Markus P. Ahola, Esa Lehikoinen, Tapio Eeva

**Affiliations:** 1 Department of Biology, University of Turku, 20014 Turku, Finland; 2 Department of Social Research, University of Turku, 20014 Turku, Finland; 3 Environmental Research and Monitoring, Swedish Museum of Natural History, 10405 Stockholm, Sweden

**Keywords:** Climate change, Population dynamics, Structural equation model, Climwin

## Abstract

How environmental factors influence population dynamics in long-distance migrants is complicated by the spatiotemporal diversity of the environment the individuals experience during the annual cycle. The effects of weather on several different aspects of life history have been well studied, but a better understanding is needed on how weather affects population dynamics through the different associated traits. We utilise 77 years of data from pied flycatcher (*Ficedula hypoleuca*), to identify the most relevant climate signals associated with population growth rate. The strongest signals on population growth were observed from climate during periods when the birds were not present in the focal location. The population decline was associated with increasing precipitation in the African non-breeding quarters in the autumn (near the arrival of migrants) and with increasing winter temperature along the migration route (before migration). The number of fledglings was associated positively with increasing winter temperature in non-breeding area and negatively with increasing winter temperature in Europe. These possible carry-over effects did not arise via timing of breeding or clutch size but the exact mechanism remains to be revealed in future studies. High population density and low fledgling production were the intrinsic factors reducing the breeding population. We conclude that weather during all seasons has the potential to affect the reproductive success or population growth rate of this species. Our results show how weather can influence the population dynamics of a migratory species through multiple pathways, even at times of the annual cycle when the birds are in a different location than the climate signal.

**Supplementary Information:**

The online version contains supplementary material available at 10.1007/s00442-020-04817-3.

## Introduction

A central ecological topic is to quantify extrinsic (e.g. weather conditions) and intrinsic (e.g. population density and breeding success) factors behind population growth rate. For long-distance migratory species, this is complicated by the diversity of environmental conditions the individuals experience during the annual cycle and along the migratory route (Fretwell 1972; Newton 1998; Sillett et al. 2000). Environmental conditions at the breeding grounds are directly related to the breeding performance and survival of both adults and offspring. Conditions in non-breeding areas and along migratory route directly affect individual survival but may also have carry-over effects on breeding success via condition and timing of reproduction (Webster et al. 2002; Norris and Taylor 2006; Sæther et al. 2006; van de Pol et al. 2010; Rushing et al. 2017). Whether it is the breeding, non-breeding, or migration phase that most affect the population growth rate varies between species and populations (Gienapp et al. 2007; Wilson et al. 2011; Rubolini et al. 2007). However, for European birds that winter in sub-Saharan Africa, it has been suggested that the weather in the non-breeding grounds may have a central role behind observed declining trends of several species (Sanderson et al. 2006; Both et al. 2006b; but see Ockendon et al. 2012). Adverse weather conditions along the migratory route may also delay arrival and decrease survival during migration with potential adverse effects on population size (Hüppop and Winkel 2006; Møller et al. 2008; Sillett and Holmes 2002; Klaassen et al. 2014).

Despite the possible carry-over effects and mortality during winter and migration, population growth ultimately depends on success of reproduction (Newton 2004; Ockendon et al. 2013). For example, the weather during the incubation and nestling phase determines individual breeding success in many avian species (Crick 2004; Pearce-Higgins et al. 2015). To study factors behind population density and the responses of the species to climate change, we thus need long-term data from the whole seasonal cycle for the species (Knudsen et al. 2011). Population growth may, for example, be density dependent, as the population size may limit the local survival or fecundity, e.g. due to increased competition for resources in breeding or non-breeding grounds (Sutherland 1996; Newton 2004). Timing of breeding is commonly related to clutch size in birds, with decreasing clutch size and nestling number for late breeding individuals (Crick et al. 1993). However, how this relates to population growth rate is not well understood, partly because it represents an indirect effect of reproductive timing on population growth rate. Modern statistical tools are thus needed to separate the direct and indirect effects of different variables. For example, the weather may directly affect breeding success or indirectly via timing of breeding. Consequently, better knowledge for the factors driving population growth are needed even for the most intensively studied migratory birds, such as the European pied flycatcher (*Ficedula hypoleuca*).

The pied flycatcher is a small insectivorous hole-nesting passerine bird that winters in non-breeding area in Africa, south of the Sahara Desert (Curry‐Lindahl 1981; Ouwehand et al. 2016). In its non-breeding grounds, it defends territories in variable habitats, from gallery forests to tree savanna, and in the summer, it breeds in open woodlands in Eurasia (Salewski et al. 2003). Previous studies have observed mixed results for factors behind breeding population density in pied flycatchers: Virolainen (1984) linked annual changes in population density to breeding success in the preceding year (see also Chernetsov et al. 2009), but Lack (1966) did not find a similar effect. Cold weather conditions on the migration route delay spring arrival times (Ahola et al. 2004, see also Laaksonen et al. 2006; Thingstad et al. 2006), but also departure date from Africa determines arrival to breeding grounds (Ouwehand and Both 2017). Delayed spring arrival may lead to mistiming the arrival in relation to food availability peak (insect abundance) with adverse effects on breeding success in pied flycatchers (Both et al. 2006a). Variable responses in previous studies partly reflect variation between populations in different parts of the species’ distribution (Both et al. 2006b). The pied flycatcher, for example, is not declining in many parts of the distribution, such as in Finland (Väisänen and Lehikoinen 2013), whereas it may show declining trends in some areas (Both et al. 2006a).

Here, we study the effects of extrinsic (climate conditions in different phases of the annual cycle and along the migratory route) and intrinsic (population density and breeding success) factors on population growth of pied flycatchers. We use data from two closely situated nest-box populations in Southwest Finland that together form an exceptionally long dataset spanning 77 years. First, we selected the climatic windows during the whole annual cycle from different parts of the migratory route that would directly best explain the population growth rate and fledgling number in our population. Second, we applied a recently developed residual dynamic structural equation modelling (RDSEM) framework that combines time-series regression modelling with structural equation modelling to separate the effects of breeding success and the identified climatic factors on the population growth rate. We predicted that adverse weather conditions on the non-breeding and migration area should have a negative impact, and good reproductive output in the previous year should have a positive impact on population growth rate. We also considered if the population growth rate was density dependent by including the population density index of the previous year in the analysis. Third, we used multilevel structural equation modelling (MSEM) to study the direct effects of weather (i.e. temperature and precipitation) during the nestling period and indirect effects of climatic factors along the migration route (via timing of breeding and clutch size) on the number of fledglings produced. We also analysed whether timing of breeding has a direct or indirect effect via clutch size on fledgling number. Our aim was to identify which climatic factors in different phases of the annual cycle most affect the population growth rate in this long-distance migratory species.

## Methods

### The study areas and data

We used breeding data from two nest-box study areas located in Southwest Finland. The first dataset was collected by Professor Lars von Haartman in area of Askainen from 1941 to 1989 (60°30′N, 21°45′E; Haartman 1951, 1960). The second dataset was collected by T.E. and E.L. in area of Harjavalta from 1991 to 2018 (61°20′N, 22°10′E, 95 km from Askainen). Both study areas are located in coniferous forest-dominated landscapes with occasional agricultural fields and rural habitation. For both datasets, the nest boxes were checked at least once a week. First, egg-laying dates were determined from the number of eggs observed during the laying period, assuming a laying frequency of one egg per day (Cramp and Perrins 1993). Clutches were considered complete when no more eggs were laid, and incubation had begun. Hatching date (i.e. the day when eggs hatch) was estimated from the wing length of small (≤ 7 days) nestlings by comparing it with growth curves of nestlings with known age (from now on we use the term “timing of breeding” to refer to hatching date). The number of fledglings was determined from the maximum number of chicks seen at the age of 10 days or older minus any dead chicks remaining in the nest after fledging. Only the first breeding attempts of a female per season were included. For more information on our datasets, please see Eeva et al. (1997), Ahola et al. (2004) and Laaksonen et al. (2006). The information on the number of nest boxes checked, breeding birds in our study areas and environmental variables included can be seen in Supplement C. On average (range), there were 113 (12–155) and 375 (293–544) available nest boxes annually, in Askainen and Harjavalta, respectively. In these nest boxes, the occupancy percentage of the pied flycatcher was 47% (range 23–83%) in Askainen and 42% (range 27–60%) in Harjavalta. In total, the data included 2379 nests in Askainen (years 1941–1994) and 4326 nests in Harjavalta (1991–2018).

**Fig. 1:**
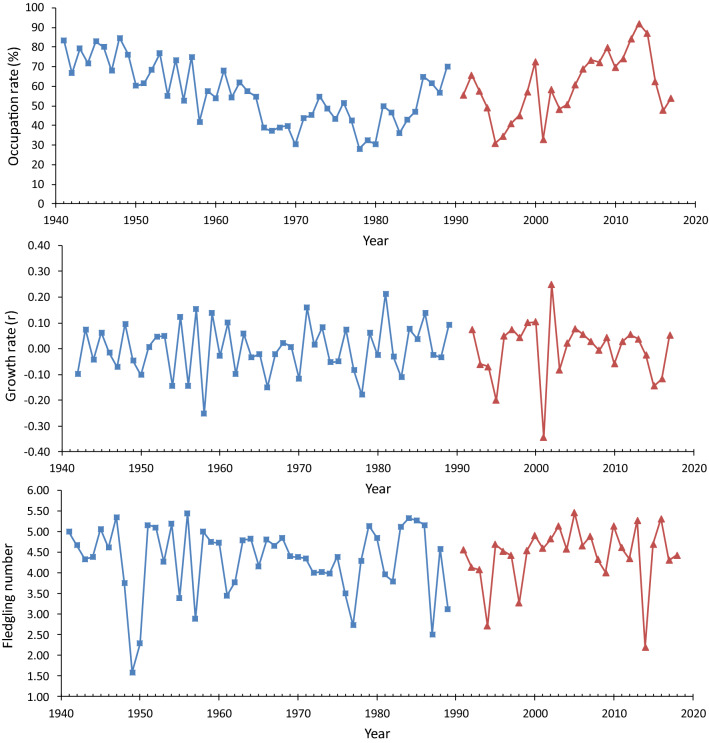
Time series for annual occupation rate (% of free nest boxes occupied), population growth rate (difference in log-abundance) and annual mean fledgling number for pied flycatchers in Askainen (blue) and Harjavalta (red) (colour figure online)

Following the earlier studies of these data (Ahola et al. 2004; Laaksonen et al. 2006), the times series from the Askainen and Harjavalta were combined because they were from different time periods but closely located spatially, and together enabled using a long time series of 77 years. For the three overlapping years (1991, 1993, and 1994), we used the values of the larger Harjavalta dataset. As an index for population density (N_t_), we used the yearly occupancy rate of available nest boxes in surveyed populations (nest boxes used by the pied flycatcher/[all surveyed nest boxes—nest boxes occupied by Parids]). Pied flycatcher occupancy rate did not correlate with Parid occupancy rate in these data (*r*_*p*_ = 0.087, *p* = 0.45, *n* = 77). Thus, availability of nest boxes for pied flycatchers did not depend on the number of Parids within the study areas. We defined population growth rate as the difference in log-abundances, calculated as *R*_t_ = log(*N*_t_) − log(*N*_t-1_), where *N*_t_ is the population density index in year_t_, and *N*_t-1_ is the density index in the previous year (Bjørnstad et al. 1999). Time series for nest-box occupation rate as a measure of population density, population growth rate and annual mean nestling number can be seen in Fig. 1. We could not control effects of breeding and natal dispersal on our population density index. However, we did not see any reason to suspect this to have major effect on our growth rate estimates.

### Weather data

**Fig. 2:**
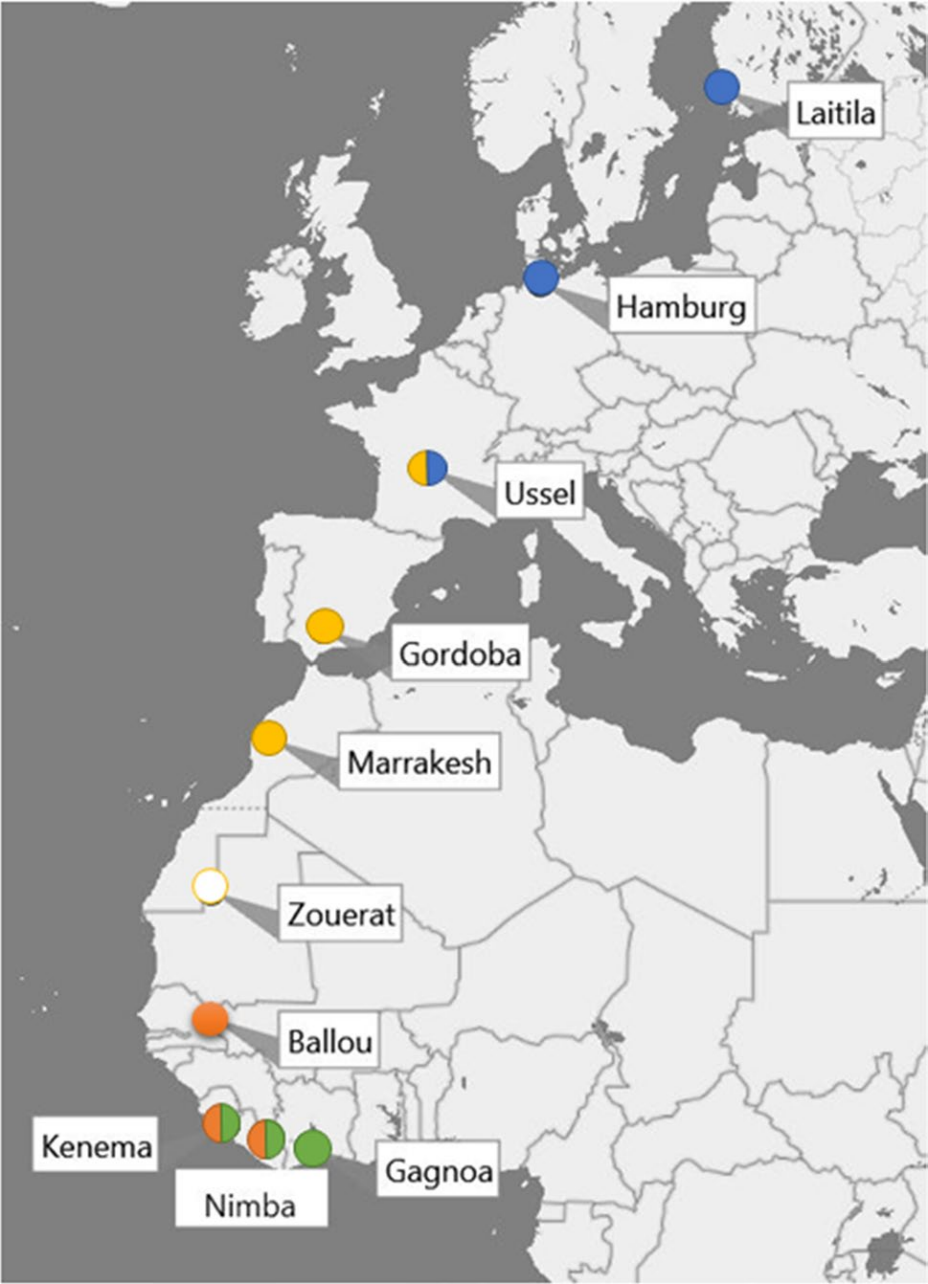
Sites (half-degree cells by Harris et al. 2014) used to measure climate during annual cycle of pied flycatchers. Laitila = breeding grounds; Hamburg–Ballou = migratory route; Kenema, Nimba and Gagnoa = non-breeding grounds. The sites within migratory route were selected based on data on recovery of ringed Finnish Pied flycatchers during migration (Valkama et al. 2015). Colours indicate climate variables composed with Climwin (supplement A) used in structural equation models: blue European region, including breeding range and northern parts of migratory range; yellow Mediterranean region within migration route; green and orange African region in non-breeding range (white not used in SEM) (colour figure online)

For temperature (monthly C°) and precipitation (mm/month) in non-breeding, migration, and breeding grounds, we used mean monthly weather data for the period 1940–2018 from Google Earth Pro (2019 Google Inc.) made available by Harris et al. (2014). We used weather data in 10 half-degree cells describing the location of pied flycatchers during their annual cycle (Fig. 2). The sites within migratory route were based on current data on recovery of ringed Finnish pied flycatchers (Valkama et al. 2015). Weather variables are spatially autocorrelated up to hundreds of kilometres, and, thus, the selected sites describe climate in larger area in different parts of migratory route and non-breeding area. For each location, we considered temperature and precipitation for the 12 months preceding breeding, that is, from July year_t-1_ (the end of previous breeding season) to June year_t_, each year. For the analysis of fledgling numbers (see below), we also used the average daily temperature and precipitation sum for the 2-week period after hatching (14 days, including the hatching date) in each nest. This time period is crucial for the survival of nestlings in this population (Eeva et al. 2002, 2020). The daily weather data were obtained from the closest weather stations maintained by the Finnish Meteorological Institute. Weather recording stations were at the same altitude as the study sites and located within 20 km from Askainen (Turku, Artukainen 1941–1954; Airport 1955–1989) and Harjavalta (Rausenkulma 1991–2009; Tulkkila 2010–2018).

### Statistical analyses

#### Locating the most relevant climate windows

To select the time period (i.e. climatic window) for each of the ten locations along the migration route (Fig. 2) that most affected the fledgling number and population growth rate each year, we used a “sliding window” approach (package Climwin in R; van de Pol et. al. 2016). This approach is used to statistically identify the best time window in which an environmental variable (here, monthly average temperature and precipitation; from now on called as climate variables) has the strongest influence on the dependent variable (population growth rate and fledgling number). The models were run altogether for 91 different time windows (for further information see Supplement A). The analysis uses the ΔAIC values to compare models with differing sliding windows to the null model without climate variables. The Climwin may suffer from overfitting and is best used as an exploratory data analysis tool (van de Pol et. al. 2016). Thus, we used the sliding window analysis only to select climate variables for the structural equation models described below.

#### Modelling population growth rate

**Fig. 3:**
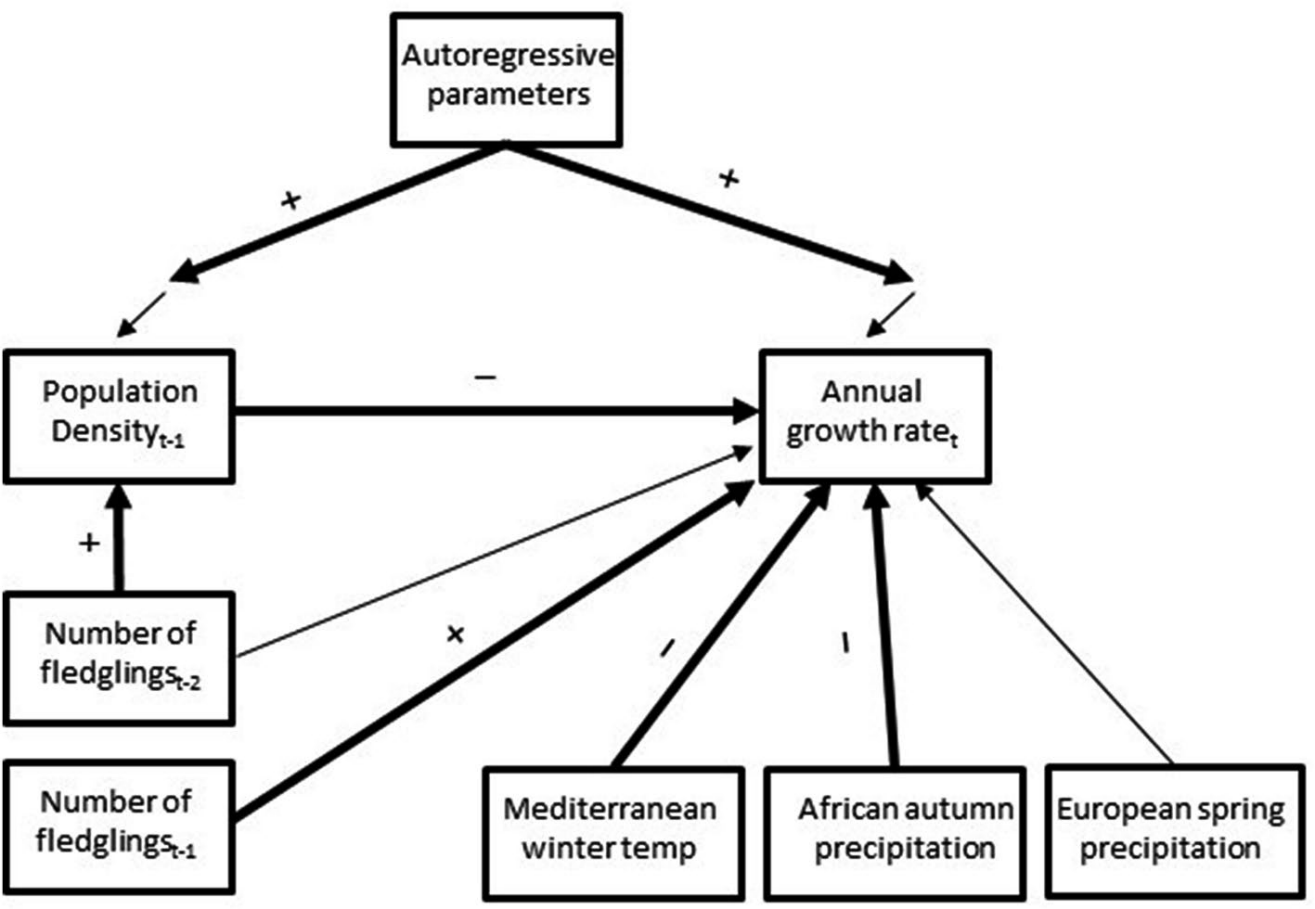
Residual dynamic structural equation model for annual population growth rate of pied flycatchers. Climate variables were indicated by a sliding window analysis. Single-headed arrows represent regression coefficients from independent variables to dependent variables (i.e. direct effects). Arrows pointing at the response variables without any relation to an observed variable represent residual errors (i.e. unobserved causes). Furthermore, arrows pointing to these residual errors denote to the potential residual autocorrelation parameters. Intercept and mean parameters of variables are omitted for simplicity. Paths that were found to be statistically non-zero are shown in bold (+ positive association, − negative association) (colour figure online)

To examine factors affecting annual population growth rate, we applied a recently developed method of residual dynamic structural equation modelling (RDSEM), which combines time series regression modelling and structural equation modelling (Aspaurohov and Muthén 2019). In this approach, the serial correlation of observations measured through time is handled by modelling the potential autocorrelation structure of the residuals of dependent variables (here, population growth rate and density index), whereas their structural associations are modelled via structural equations. In other words, this approach is capable of modelling direct and indirect effects while accounting for the potential non-independence of residuals in all equations. The model fitted is shown in Fig. 3. It depicts how selected climatic windows are assumed to affect the annual population growth rate. The annual population growth rate is further assumed to be associated with the average number of fledglings and population density index in the previous year, as well as the average number of fledglings two years apart (some individuals may enter the breeding population only at the age of 2 years) (Fig. 3). We also included potential autocorrelation structures of the residuals of the average fledgling number from the previous year and annual population growth rate by fitting autoregressive terms (AR(*n*), where *n* denotes to the length of lag) for those residuals up to a lag of 3 years. If not differing statistically from zero (c.l. overlapping with 0), the autoregressive terms were dropped from the final model.

#### Modelling fledgling number

**Fig. 4:**
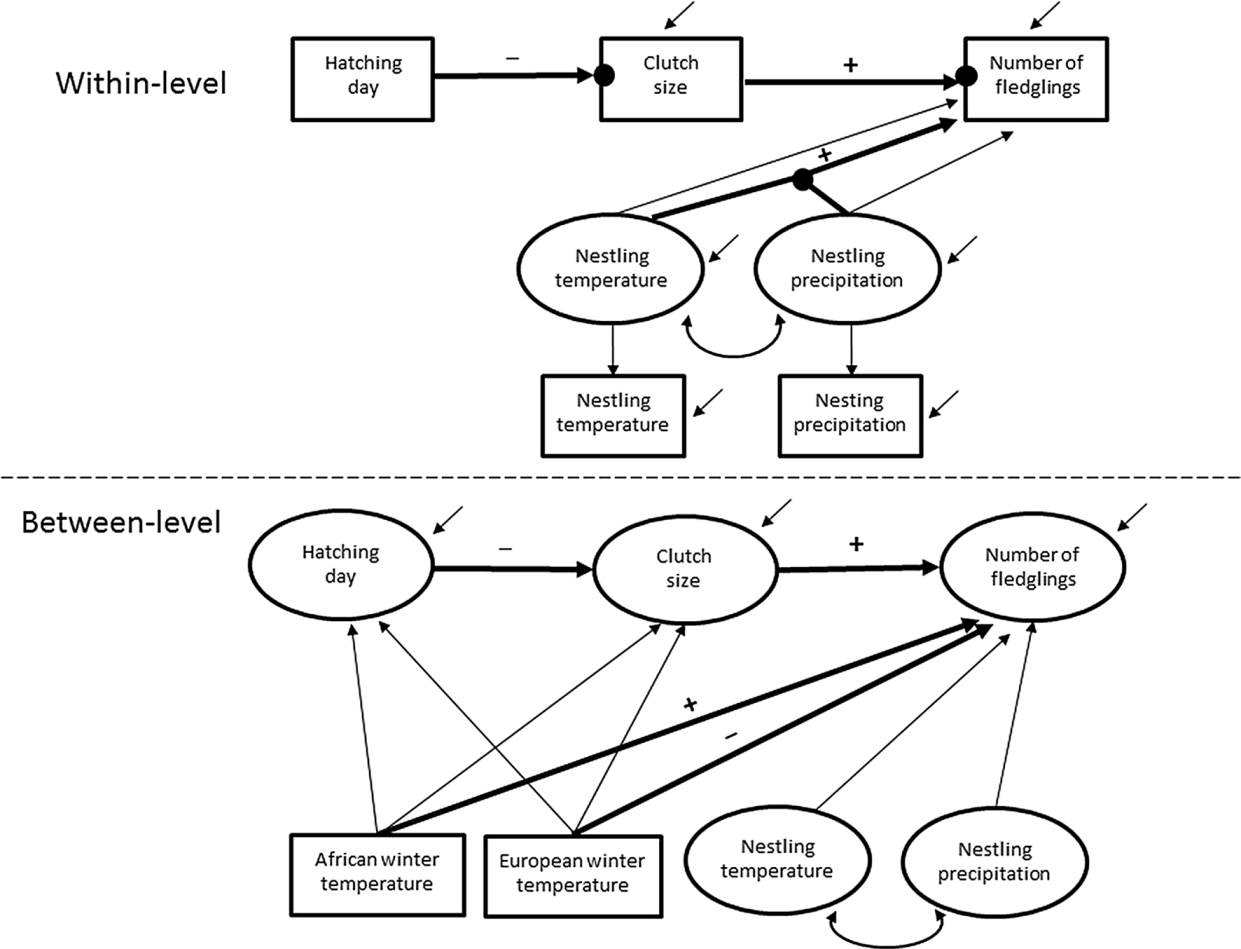
Multilevel structural equation modelling looking at how selected climate variables (sliding window analysis) influenced the number of fledglings directly and indirectly via hatching day and clutch size between the years (the between-level). At the within-year level (among individual nests), the number of fledglings was influenced by climate during the nestling period as well as their interaction. Observed variables are represented as boxes and unobserved latent variables as circles. In multilevel models, the within-level response variables have random intercepts at the between-level that are continuous latent variables and vary across clusters (i.e. years). At the within-level, this is represented as filled circles at the end of arrows from independent variables to dependent variables. At the between-level, these random intercepts are regressed onto between-level independent variables. Moreover, at the within-level, the arrow originating from the connecting dot between breeding temperature and precipitation represent their interaction influencing the number of fledglings. Open short arrows denote to residual variances of dependent variables. Paths that were found to be statistically non-zero are shown in bold (+ positive association, − negative association)

Using data on individual nests, we examined how the selected climatic windows influenced the number of fledglings directly and indirectly via clutch size and hatching day. Average daily temperature and precipitation sum during the nest-specific nestling periods (2 weeks from hatching) were also included in the model. We used a multilevel structural equation model enabling both within- and between-year associations (Preacher et al. 2010; Fig. 4). The climatic windows defined by the Climwin analysis (the temperature in winter in Europe and Africa, Table S1) were monthly average values and, thus, could be compared only between the years. Instead, the data for individual nests (temperature and precipitation during nestling period, hatching day, clutch size and our outcome, the number of fledglings) were measured within the years allowing analysis both within- and between-year levels. Further, as can be seen from Fig. 4, the total association of predictors to the outcome can be decomposed into direct and indirect effects: clutch size has direct influences only whereas hatching day and climatic windows have both direct and indirect influences (total effect is a sum of direct and indirect effects). Moreover, the effect of temperature during nestling period could be moderated by precipitation during nestling period (i.e. there can be an interaction in the effect of these two variables). Climatic windows act at the between-level, hence their indirect influences on the outcome are dealt with at the between-level (Preacher et al. 2010).

Please note that it was not possible to combine the two models since our main outcome, population growth rate, was measured annually, making it impossible to estimate indirect effects for within-year predictors used in the current model (Preacher et al. 2010). Both models were analysed with Bayesian estimation using Mplus 8.4. For further information, see Supplement B.

## Results

### Factors affecting population growth rate

The climate window analyses identified three climate windows during the preceding non-breeding season that appeared to be associated with population growth rate (supplement A Table S1): winter temperature in the Mediterranean region (average for monthly values of December and January), precipitation in Africa during the previous autumn (orange locations in Fig. 2; monthly averages September–October), and spring precipitation in Europe (January–April). We used these windows to form three climate variables that were with other variables simultaneously included in the RDSEM model for testing their effects on the population growth rate.

Of these climatic variables, population growth rate was directly associated with the winter temperature in the Mediterranean region and rainfall in Africa during the previous autumn (Table 1; Fig. 3). A 1-degree (°C) increase in the average winter temperature in the Mediterranean region decreased the population growth rate by 0.025 units (the unit is a difference in log-abundance, compare to supplement A Fig. S2), whereas a 1-mm increase in the average autumn precipitation in Africa decreased the population growth rate by 0.0007 log-difference units (Table 1). Spring precipitation in Europe was not significantly associated with annual population growth rate in these data (Table 1).

**Table 1:** Results of residual dynamic SEM for (log) population growth rate of pied flycatchers

Structural parameters	Median	95% Credibility intervals	
		Lower 2.5%	Upper 2.5%
Annual population growth rate			
Population density index_t-1_	**− 0.003**	**− 0.005**	**− 0.002**
Number of fledglings_t-1_	**0.028**	**0.005**	**0.051**
Number of fledglings_t-2_	**− **0.004	**− **0.026	0.018
Mediterranean winter temperature	**− 0.025**	**− 0.045**	**− 0.006**
European spring precipitation	**− **0.00161	**− **0.00354	0.00025
African autumn precipitation_t-1_	**− 0.00066**	**− 0.00120**	**− 0.00013**
AR(2)	**0.415**	**0.126**	**0.692**
Population density index			
Number of fledglings_t-2_	**4.34**	**1.82**	**6.90**
AR(1)	**0.565**	**0.324**	**0.804**
AR(2)	**0.257**	**0.015**	**0.499**
Means			
Number of fledglings_t-1_	4.359	4.168	4.550
Number of fledglings_t-2_	4.363	4.171	4.560
Intercepts			
Annual population growth rate	0.093	**− **0.072	0.256
Population density index_t-1_	40.673	23.210	60.409
Variances			
Number of fledglings_t-1_	0.702	0.495	0.960
Number of fledglings_t-2_	0.715	0.503	0.980
Residual variances			
Annual population growth rate	0.006	0.004	0.008
Population density index_t-1_	115.68	80.61	159.83

See Fig. 3 for model structure. Parameters statistically differing from zero are shown in bold

An increase of one fledgling in the average fledgling number in the previous year increased the annual population growth rate by 0.028 units (Table 1). The average fledgling number 2 years prior was neither directly associated with annual population growth rate (Table 1) nor did its total effect differ statistically from zero (median = − 0.019, 95% CIs = − 0.042, 0.003). Its indirect effect via the following year’s population density on annual population growth rate was, however, negative (median = − 0.014, 95% CIs = − 0.028, − 0.004; note that this was the only variable separated into indirect and direct effects in this model, see Fig. 3). The effect of the population density index (i.e. nest-box occupation rate) suggested density dependence, as a one-unit increase in the previous year’s population density index decreased population growth rate by 0.003 log-difference units. We also identified second- and first-order autoregressive processes for the residuals of population density (Table 1), suggesting that population density positively correlated with the density of the previous 2 years. A second-order autoregressive process was also found for annual population growth rate (Table 1).

### Factors affecting number of fledglings

#### Between-year effects

At the between-year level, average monthly winter temperatures in Europe (February) and Africa (November; the climatic windows selected for this model, supplement A Table S1) were directly associated with the average number of fledglings produced yearly (Tables 2 and 3, Fig. 4). A 1-degree (°C) increase in the average winter temperature in Europe decreased the average number of fledglings by 0.071 fledglings (Table 2). In contrast, a 1-degree (°C) increase in average winter temperature in Africa increased the average number of fledglings by 0.67 fledglings (Table 2, supplement A Fig. S2). No statistically non-zero indirect effects of winter temperatures in Europe or Africa, via timing of breeding and clutch size or both, on average number of fledglings were found (Table 3). Yet, these indirect effects strengthened the total effects of European and African winter temperatures on annual fledgling numbers (− 0.089 and 0.726 for European and African winter temperatures, respectively, see Table 3). Neither average temperature nor precipitation during the nestling period of the population was associated with the average number of fledglings (Table 2).

**Table 2:** Result of a multilevel SEM examining within-years (within-level) the direct effect of clutch size and climate during nestling period on fledgling number, and the effect of hatching date on clutch size. At the between-level, the same effects are estimated as well as the effect of European and African winter temperatures on hatching date, clutch size and fledgling number

		Posterior median	95% Credibility intervals	
			Lower 2.5%	Upper 2.5%
Within-level				
Structural parameters				
Number of fledglings				
Nestling temperature		− 0.009	− 0.059	0.041
Nestling precipitation		− 0.002	− 0.006	0.002
Temperature × precipitation		**0.0044**	**0.0006**	**0.008**
Clutch size		**0.695**	**0.635**	**0.755**
Clutch size				
Hatching date		− **0.058**	− **0.062**	− **0.053**
Covariances				
Breeding temperature with precipitation		− 0.023	− 0.026	− 0.019
Variances				
Breeding temperature		0.994	0.955	1.034
Breeding precipitation		16.02	15.39	16.6
Hatching date		2.35	2.26	2.45
Residual variances				
Number of fledglings		2.98	2.86	3.10
Clutch size		0.58	0.56	0.60
Between-level				
Structural parameters				
Number of fledglings				
Clutch size		**1.489**	**0.567**	**2.409**
Nestling temperature		0.058	− 0.007	0.124
Nestling precipitation		0.006	− 0.001	0.013
European winter temperature		− **0.071**	− **0.121**	− **0.021**
African winter temperature		**0.670**	**0.355**	**0.979**
Clutch size				
Hatching date		− **0.037**	− **0.050**	− **0.025**
European winter temperature		− 0.013	− 0.028	0.001
African winter temperature		0.019	− 0.073	0.110
Hatching date				
European winter temperature		− 0.003	− 0.031	0.024
African winter temperature		− 0.048	− 0.222	0.128
Means				
Nestling temperature		15.11	14.70	15.54
Nestling precipitation		27.35	23.30	31.34
Intercepts				
Number of fledglings		− 5.55	− 11.51	0.52
Clutch size		8.89	8.05	9.76
Hatching date		69.07	68.41	69.74
Covariances				
Nestling temperature with precipitation		− 0.057	− 0.142	0.021
Variances				
Nestling temperature		3.46	2.43	4.76
Nestling precipitation		30.99	21.69	42.49
Residual variances				
Number of fledglings		0.166	0.095	0.254
Clutch size		0.011	0.005	0.019
Hatching date		0.80	0.54	0.111

See Fig. 4 for model structure. Parameters statistically differing from zero are shown in bold

**Table 3:** Between-level total effects of winter temperature in Europe and Africa and hatching date on the annual number of fledglings, decomposed into direct and indirect effects, where indirect effects are further divided into effect via hatching date and clutch size

	Winter temperature	Winter temperature	Hatching
	in Europe	in Africa	date
Total effect	− 0.089 (− 0.143, − 0.036)	0.726 (0.390, 1.064)	− 0.055 (− 0.093, − 0.019)
Direct effect	− 0.071 (− 0.121, − 0.021)	0.670 (0.355, 0.979)	–
Indirect effect	− 0.016 (− 0.049, 0.011)	0.050 (− 0.122, 0.250)	− 0.055 (− 0.093, − 0.019)
Via hatching date	0.002 (− 0.015, 0.019)	0.023 (− 0.077, 0.140)	–
Via clutch size	− 0.018 (− 0.047, 0.003)	0.025 (− 0.112, 0.185)	− 0.055 (− 0.093, − 0.019)

95% CI of the posterior median in parentheses

An increase of one egg in average annual clutch size directly increased the average fledgling number by 1.49 fledglings (Table 2). This means that in years with larger clutch sizes, the fledgling production was disproportionately good (or vice versa). Moreover, a delay in timing of breeding by 1 day resulted in a decrease of 0.037 eggs in average clutch size (Table 2). The total effect of average timing of breeding on average number of fledglings was − 0.055 (Table 3).

#### Within-year effects

**Fig. 5:**
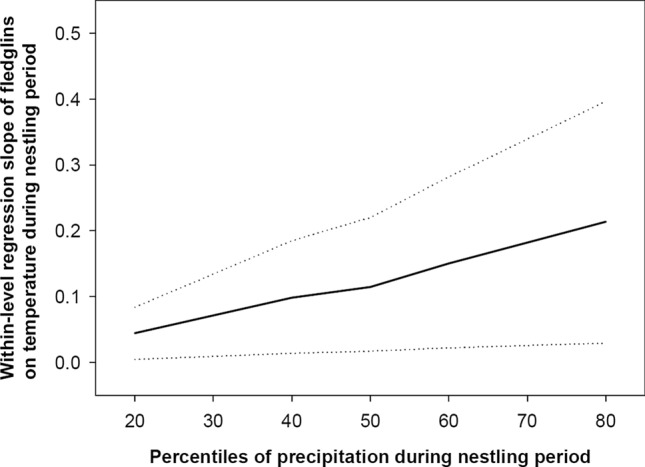
Within-level regression slope of the number of fledglings on temperature during nestling period (regression slopes as a solid line and, 95% CI as dotted lines), given for different percentiles of the distribution of nestling period precipitation. That is, the positive effect of temperature on fledgling number was higher when there was more precipitation

At the within-year level, we found that the influence of temperature during nestling period on the number of fledglings was moderated by the amount of precipitation during the same period (i.e. an interaction between nestling period temperature and precipitation; Table 2). That is, the positive effect of temperature got stronger with the increasing precipitation during the nestling period (Fig. 5). In this within-year model, an increase of one egg in clutch size increased the number of fledglings by 0.695 fledglings, while a 1-day delay in timing of breeding decreased clutch size by 0.058 eggs (Table 2). The total effect of hatching day on the number of fledglings was − 0.040 (95% CIs = − 0.045, − 0.035; Table 3).

## Discussion

We examined in two different steps how climatic factors along different parts of the annual cycle and migratory route affect population growth rate (the change in breeding population density) in a long-distance migrant, the pied flycatcher. In the first step, we found that the population growth rate was associated with climatic factors on the migration route and non-breeding grounds during the year between the two breeding seasons. Interestingly, the strongest signals were from climate during periods of the annual cycle when the birds were not present in the focal location. The population decline was associated with increasing winter temperature along the migration route in the Mediterranean region (before the birds migrate through to north in the spring) and with increasing rain in the African winter quarters in the autumn (before or near the arrival of the birds). High population density and low fledging production were the intrinsic factors reducing the size of the next year’s breeding population. In the second step, we, therefore, examined the climatic factors affecting the number of fledglings produced during the breeding season. The number of fledglings produced in our northern European population was associated positively with increasing temperature during the winter in Africa (i.e. half a year before the breeding season) and negatively with increasing temperature in central Europe in the winter (when the birds are still in Africa). The multilevel structural equation model that we used allowed us to verify that these possible carry-over effects did not arise via timing of breeding or clutch size but were rather direct effects on the number of fledglings. The climate during the breeding season affected reproductive output as well, with a positive effect of high temperature being more pronounced with higher precipitation. Altogether, our results show how weather can influence the population dynamics of a migratory species through multiple pathways, even at times of the annual cycle when the birds are in a different location than the climate signal.

### The effect of climatic factors on migratory route and non-breeding grounds

Pied flycatchers are rarely present in Europe in winter (some individuals may overwinter in the Mediterranean region). Still, the winter temperature in the Mediterranean region (or in southern Europe in Spain and France, see Table S1) had a negative influence on population growth rate, and the winter temperature in Europe (most strongly close to Germany) had negative influence on fledgling number. It is clear that these associations cannot indicate any direct effect on pied flycatcher individuals, but have to arise through some correlated factors. Based on our data, we can only speculate what these factors could be. For example, the effect of winter weather can affect the abundance of insects which are used as food later in spring when pied flycatchers are present in the area. For herbivorous insects, it is known that the temperature is a dominant abiotic factor affecting populations, the major effect in temperate regions arising via decreased winter survival in rising temperatures (Bale et al. 2002; Salis et al. 2016), which can be reflected in insect numbers later in spring and summer (Harris et al. 2019). Stopovers during migration are shorter in spring than in autumn in pied flycatchers (Valkama et al. 2015), but it is clear that they cannot survive without food for 2–3 weeks that is typical length of spring migration in the species (Ouwehand et al. 2016). In addition, the first stopovers after migrating over the Sahara Desert and the Mediterranean Sea typically are very important for the survival of European long-distance migrants (Klaassen et al. 2014; Arlt et al. 2015). In our case, a 4-degree (ºC) change in Mediterranean winter temperature, which is within the limits of temperature change observed during our study period, would result in a 0.1-unit change in population growth rate (which varies between values 0.25 to − 0.35, see supplement Figure S2). This kind of change would have a substantial effect on the population size of the species. However, to verify how winter weather in Europe affects pied flycatchers, we need further experimental studies. Our data was, for example, at very coarse level using monthly average climate data and finer scale analyses may be needed to understand the mechanisms behind the observed patterns (Haest et al. 2019).

Population growth rate was also linked to the autumn precipitation in non-breeding areas in Africa. This relationship was negative, in contrast to some earlier long-distance migrant studies where precipitation in non-breeding areas in Africa has been observed to have positive effects (e.g. Peach et al. 1991; Mihoub et al. 2010; García‐Pérez et al. 2014; Rockwell et al. 2017). However, rainfall is particularly important in arid non-breeding areas (Blendinger and Ojeda, 2001; Smith et al. 2010; Tøttrup et al. 2012), whereas pied flycatchers’ winter in moist areas south of the Sahel (Ouwehand et al. 2016). In our case, the timing of the effect of precipitation in Africa was close to the arrival of birds to the region. Thus, one possibility is that very rainy conditions in the non-breeding area at the time when the pied flycatchers arrived from autumn migration had adverse effects on survival. This remains speculation, and the observed negative correlation was also relatively weak in the current analysis.

A positive association between temperature in non-breeding areas and fledgling production was very clear in our analysis. Previous studies assessing the role of temperature in non-breeding areas to the components of population size remain relatively few for long-distance migrants. For example, Gordo et al. (2005) observed that increased temperature in non-breeding areas delayed common nightingale arrival to breeding areas that lead to a reduction in fledgling numbers. In the case of the barnacle goose, non-breeding temperature was found to be positively related to brood size (Cabot and West 1973). However, Finch et al. (2014) did not observe any effect of non-breeding temperature on breeding success of three passerine species (see also Pearce-Higgins and Green 2014; Mondain-Monval et al. 2019). In our case, the mechanism behind the observed association between temperature of the non-breeding grounds, and nestling production later in the breeding grounds, remains unclear. The observed association was direct and not, e.g. acting via timing of breeding. Perhaps the low temperature in non-breeding areas might, for example, be related to disease prevalence or some other environmental condition that decreases the condition of parent birds, which then carries over to breeding success (Marra et al. 1998). Since fledgling production was found to be an important determinant of the population growth rate, factors affecting it will also ultimately affect the population size.

Weather during migration is commonly observed to affect timing of spring arrival in birds (Norris and Marra 2007; Knudsen et al. 2011; Charmantier and Gienapp 2014), but timing of breeding may also depend on timing of departure from non-breeding area (Ouwehand and Both 2017). These effects were not studied here, but timing of breeding clearly influenced clutch size in the current study. If we compare the effect of timing of breeding to the observed climate effects on number of fledglings in the current analysis, the 10-day change in timing of breeding produces comparable effects as a 5-degree (°C) change in monthly average winter temperature in Europe, or a 1-degree (°C) change in monthly average temperature at the non-breeding grounds. All the climate effects observed in the current study were direct effects and not indirect effects via timing of breeding on nesting success.

### The effect of climatic factors on breeding grounds and intrinsic factors

A high number of fledglings produced in the previous breeding season resulted in population growth, and as expected (see, e.g. Eeva et al. 2002; Burgess 2014), one determinant of fledgling number was the weather during the breeding period. In previous studies, the positive effects of breeding season temperature have been speculated to be mediated through increased invertebrate food abundance and availability, reduced thermoregulatory requirements and increased foraging time associated with warm weather (Pearce-Higgins and Green 2014). The novelty in our study is our approach to separate the within-year and between-year effects. Surprisingly, neither precipitation nor temperature affected the average annual fledgling number of pied flycatchers as a between-year effect. Instead, within-years, rain during the nestling period had a negative effect on fledging number, and this effect became stronger at cold temperatures (see also Eeva et al. 2020). The relative role of breeding season weather compared to non-breeding and migration time weather is, however, difficult to judge from our analysis. For example, the weather during breeding in Finland is more variable than the very constant winter temperature in non-breeding areas of pied flycatchers, which usually varied only within 2 °C in our data. In addition, the length of climatic windows used varied depending on location. Although non-breeding temperature was observed to have the greatest effect size, it is, thus, difficult to compare its actual influence on pied flycatcher nesting success to that of temperature during breeding or weather affecting the timing of breeding.

Density dependency is often observed in population fluctuations of avian species (Both 1998; Newton 2004; but see, Morrissette et al. 2010). In our pied flycatcher population, the population density in year_t_ was negatively related to population density in year_t-1_, even though we controlled for the climate variables during the annual cycle and for the breeding success in the previous year. This meant that a 10% increase in density in the previous year resulted in a decrease in population growth rate by 7%. Competition between individuals that decreases survival is one possible mechanism behind these patterns (Newton 2004). There was also positive autocorrelation with the 2-year time lag in population growth rate, indicating that the population had some periods of growth or decline that lasted over several years. Finally, we also found the interesting pattern that, in between-years, the effect of clutch size was disproportional on the number of fledglings; that is, in the years of large clutch sizes, the fledgling production was disproportionately large (or in years of small clutch sizes the fledgling production was disproportionately weak). In the analysis within-years, no similar association was detected. This indicates that low average clutch sizes in certain years might indicate general low condition/quality of females in those years, resulting in even lower nestling numbers.

### Conclusions

We conclude that climate during all seasons, at breeding, migration and non-breeding grounds, has the potential to affect the breeding success and population growth rate of the pied flycatcher breeding in Southwest Finland. This highlights how complex it may be to predict the responses to climate change for migratory species. We assert that for population dynamics of migrants, it is important to consider the weather in regions where individuals are currently not located. Similarly as observed, for example, for migrating ungulates, for which winter weather that determines plant growth in spring ranges of the species has major influence on reproductive success (Post and Forchhammer 2008, see also Selonen et al. 2020). It is also well known that weather has a major influence on population fluctuations of a wide group of different organisms (Post and Forchhammer 2002; Sheppard et al. 2015). In addition, even small long-term changes in the climate that affect population growth rate may have major consequences for the population size over time (Saether et al. 2000). In the current study, we have been able to reveal some of the associations that potentially determine the population size of the pied flycatchers in the face of a changing climate. Our study supports the conclusion that the carry-over effects from non-breeding areas and during migration contribute to nesting success and ultimately to population growth rate of long-distance migrant passerines.

## Supplementary Information

Below is the link to the electronic supplementary material.

Supplementary file1 (DOCX 661 KB)
