# Supplementary material for: Identifying the paths of climate effects on population dynamics: dynamic and multilevel structural equation model around the annual cycle
Source: Oecologia. 2021 Jan 18;195(2):525–38. doi: 10.1007/s00442-020-04817-3 (PMC7882558; doi:10.1007/s00442-020-04817-3)
Supplement: Supplementary file 1 — Supplementary file1 (DOCX 661 KB) [file 442_2020_4817_MOESM1_ESM.docx]

**Supplement A for Selonen et al.**

*Climwin analysis*

We used Climwin package in R to select the climate variables (Bailey & van de Pol, 2016; van de Pol et. al., 2016). First, we compared candidate models with differing sliding windows to a baseline model without any climate effects included (i.e. the “null model”). In our case, to achieve stationary series, this baseline model included year for fledgling number and year and AR(1) (i.e., an autoregressive parameter for the influence of the previous year’s value) for population growth rate as predictors. The candidate models were run for all possible time windows (altogether 91 different time windows). Best models were selected based on the highest ΔAIC values that measure the difference in AIC between the candidate and the null model. To analyse whether the climate signals deviate from the null model the Climwin performs randomizations (100 rounds) of the data (Bailey & van de Pol, 2016). Linear relationships performed better than quadratic ones (lower AIC values in all cases), and the former was used for all the analyses. The descriptive metric used in sliding windows for monthly climate variables in sliding windows was mean (average of months included in the window), because minimum (month with lowest value) or maximum (month with highest value) did not perform better (ΔAIC < 2). Missing years (1990 and years before start of time series needed to fit autoregressive parameters) were imputed to model using Kalman smoother method to be able to use continuous time series for the analysis. If the timing and direction of climate signals was similar in several nearby locations, they were combined for further analysis, as shown in Table S1. This is reasonable because monthly climate variables show spatial autocorrelation over a relatively large area (e.g., Halkka et al., 2011).

Bailey, L. D., & van de Pol, M. (2016) *climwin*: An R toolbox for climate window analysis. *PLoS ONE*, 11, e0167980.

Halkka, A., Lehikoinen, A. & Velmala, W. (2011) Do long-distance migrants use temperature variations along the migration route in Europe to adjust the timing of their spring arrival? *Boreal Environmental Research*, 16, 35–48.

van de Pol, M., Bailey, L.D., McLean, N., Rijsdijk, L., Lawson, C.R. & Brouwer, L. (2016) Identifying the best climatic predictors in ecology and evolution. *Methods in Ecology and Evolution*, 7, 1246–1257.

| **Table S1.** Study locations and best-fit (largest delta AIC) climate windows (CW; 1 = January, 2 = February etc.) from linear regressions^1^ between population parameters (population growth rate and average annual fledgling number) of the pied flycatcher (*Ficedula hypoleuca*) and climate variables in 10 locations at breeding, migration and non-breeding grounds. The significance of climate signals, that is, the probability of the model to deviate from to the null model is given in the column P_dAICc_. The column FhM indicates the months when the flycatchers are supposed to be in the given location based on the migration schedule of the Finnish Bird Ringing Atlas^2^. Similar climatic signals from several nearby locations were used to build new composite variables for structural equation models (SEM). N = 77 years. | | | | | | | | | | | |
| --- | --- | --- | --- | --- | --- | --- | --- | --- | --- | --- | --- |
| Location | Country | Lat °N | Lon °E | Climate  variable | Response  variable^3^ | FhM | CW | Beta | dAIC | P_dAICc_ | Composed  variable  for SEM^4^ |
| Laitila | Finland | 60.75 | 21.75 | Temperature | Growth rate | 5-8 | 2 | 0.006 | -2.51 | 0.17 | - |
| Hamburg | Germany | 53.75 | 9.75 | Temperature | Growth rate | 8, 4-5 | 3-4 | 0.021 | -4.55 | 0.11 | - |
| Ussel | France | 45.75 | 2.25 | Temperature | Growth rate | 8, 4-5 | 12-1 | -0.034 | -10.4 | **0.01** | Mediterranean winter T  (Dec - Jan) |
| Cordoba | Spain | 37.75 | -4.75 | Temperature | Growth rate | 9, 4 | 12-1 | -0.041 | -7.53 | **0.04** |  |
| Marrakesh | Morocco | 31.75 | -8.25 | Temperature | Growth rate | 9, 4 | 1 | -0.031 | -4.12 | 0.19 |  |
| Zouerat | Mauritania | 22.75 | -12.25 | Temperature | Growth rate | 10, 4 | 10 | 0.051 | -9.96 | **0.01** | - |
| Ballou | Senegal | 14.75 | -12.25 | Temperature | Growth rate | 10-3 | 10-11 | 0.040 | -2.10 | 0.34 | - |
| Kenema | Sierra Leone | 7.75 | -11.25 | Temperature | Growth rate | 10-3 | 3 | -0.037 | -0.09 | 0.72 | - |
| Nimba | Liberia | 6.75 | -8.75 | Temperature | Growth rate | 10-3 | 1-4 | -0.040 | 0.52 | 0.82 | - |
| Gagnoa | Ivory Coast | 6.25 | -5.75 | Temperature | Growth rate | 10-3 | 1-3 | -0.044 | -0.40 | 0.64 | - |
| Laitila | Finland | 60.75 | 21.75 | Temperature | Fledglings | 5-8 | 2 | -0.071 | -6.45 | 0.13 | European  winter T  (Feb) |
| Hamburg | Germany | 53.75 | 9.75 | Temperature | Fledglings | 8, 4-5 | 2 | -0.123 | -10.13 | **0.03** |  |
| Ussel | France | 45.75 | 2.25 | Temperature | Fledglings | 8, 4-5 | 1-3 | -0.243 | -6.45 | 0.07 |  |
| Cordoba | Spain | 37.75 | -4.75 | Temperature | Fledglings | 9, 4 | 1-3 | -0.380 | -4.68 | 0.18 | - |
| Marrakesh | Morocco | 31.75 | -8.25 | Temperature | Fledglings | 9, 4 | 2 | -0.137 | -0.35 | 0.80 | - |
| Zouerat | Mauritania | 22.75 | -12.25 | Temperature | Fledglings | 10, 4 | 9 | 0.433 | -2.56 | 0.33 | - |
| Ballou | Senegal | 14.75 | -12.25 | Temperature | Fledglings | 10-3 | 11 | 0.315 | -2.48 | 0.32 | - |
| Kenema | Sierra Leone | 7.75 | -11.25 | Temperature | Fledglings | 10-3 | 11-1 | 0.840 | -6.40 | 0.07 | African  winter T  (Nov) |
| Nimba | Liberia | 6.75 | -8.75 | Temperature | Fledglings | 10-3 | 11 | 0.828 | -8.78 | **0.02** |  |
| Gagnoa | Ivory Coast | 6.25 | -5.75 | Temperature | Fledglings | 10-3 | 11 | 0.769 | -7.62 | **0.04** |  |
| Laitila | Finland | 60.75 | 21.75 | Rainfall | Growth rate | 5-8 | 3-4 | -0.0021 | -1.72 | 0.63 | European  spring R  (Jan-Apr) |
| Hamburg | Germany | 53.75 | 9.75 | Rainfall | Growth rate | 8, 4-5 | 4 | -0.0010 | -1.79 | 0.62 |  |
| Ussel | France | 45.75 | 2.25 | Rainfall | Growth rate | 8, 4-5 | 1-4 | -0.0021 | -10.5 | **0.02** |  |
| Cordoba | Spain | 37.75 | -4.75 | Rainfall | Growth rate | 9, 4 | 4-5 | 0.0010 | -1.53 | 0.60 | - |
| Marrakesh | Morocco | 31.75 | -8.25 | Rainfall | Growth rate | 9, 4 | 7 | -0.0233 | -1.97 | 0.46 | - |
| Zouerat | Mauritania | 22.75 | -12.25 | Rainfall | Growth rate | 10, 4 | NA | NA | NA | NA | - |
| Ballou | Senegal | 14.75 | -12.25 | Rainfall | Growth rate | 10-3 | 10-2 | -0.00368 | -2.09 | 0.42 | African  autumn R  (Sep-Oct) |
| Kenema | Sierra Leone | 7.75 | -11.25 | Rainfall | Growth rate | 10-3 | 10 | -0.00047 | -4.29 | 0.32 |  |
| Nimba | Liberia | 6.75 | -8.75 | Rainfall | Growth rate | 10-3 | 9-10 | -0.00071 | -1.30 | **0.01** |  |
| Gagnoa | Ivory Coast | 6.25 | -5.75 | Rainfall | Growth rate | 10-3 | 4 | -0.00069 | -4.48 | 0.27 | - |
| Laitila | Finland | 60.75 | 21.75 | Rainfall | Fledglings | 5-8 | 8 | 0.011 | -6.18 | 0.12 | - |
| Hamburg | Germany | 53.75 | 9.75 | Rainfall | Fledglings | 8, 4-5 | 4 | -0.0084 | -0.53 | 0.98 | - |
| Ussel | France | 45.75 | 2.25 | Rainfall | Fledglings | 8, 4-5 | 3 | 0.0079 | -5.24 | 0.19 | - |
| Cordoba | Spain | 37.75 | -4.75 | Rainfall | Fledglings | 9, 4 | 3 | 0.0074 | -6.21 | 0.10 | - |
| Marrakesh | Morocco | 31.75 | -8.25 | Rainfall | Fledglings | 9, 4 | 7-12 | 0.019 | -2.09 | 0.50 | - |
| Zouerat | Mauritania | 22.75 | -12.25 | Rainfall | Fledglings | 10, 4 | 1 | 0.090 | -1.14 | 0.55 | - |
| Ballou | Senegal | 14.75 | -12.25 | Rainfall | Fledglings | 10-3 | 10-5 | 0.057 | -5.27 | 0.09 | - |
| Kenema | Sierra Leone | 7.75 | -11.25 | Rainfall | Fledglings | 10-3 | 1-2 | 0.024 | -4.82 | 0.17 | - |
| Nimba | Liberia | 6.75 | -8.75 | Rainfall | Fledglings | 10-3 | 8-3 | 0.014 | -4.76 | 0.23 | - |
| Gagnoa | Ivory Coast | 6.25 | -5.75 | Rainfall | Fledglings | 10-3 | 8-3 | 0.010 | -1.75 | 0.66 | - |
| ^1^ Linear regression models with Climwin package in R. Climate variables (monthly mean temperature [°C] or rainfall [mm]), their 1^st^ order autoregressive term and year were used as explanatory factors. See van de, Pol, M., Bailey, L.D., McLean, N., Rijsdijk, L., Lawson, C.R. and Brouwer, L. 2016: Identifying the best climatic predictors in ecology and evolution. Methods Ecol Evol, 7: 1246-1257.  ^2^ Valkama, J., Saurola, P., Lehikoinen, A., Lehikoinen, E., Piha, M., Sola, P., Velmala, W., Forsman, D., Sulonen, H. 2015: The Finnish Bird Ringing Atlas, Volume II. Finnish Museum of Natural History, Helsinki. 784 pages.  ^3^ Response variables were population growth rate (log[N_t_] – log[N_t-1_]), where N_t_ is population density index in year t, and N_t-1_ is density index in the previous year) and mean annual fledgling number. Population density index denotes the proportion of nest boxes occupied by flycatchers from all available nest boxes.  ^4^ Composed new variables were used in SEM-models to explore relationships between climate variables and population parameters. | | | | | | | | | | | |

| Laitila, FL vs. Temperature | Hamburg, FL vs. Temperature | Ussel, FL vs. Temperature |  |
| --- | --- | --- | --- |
| 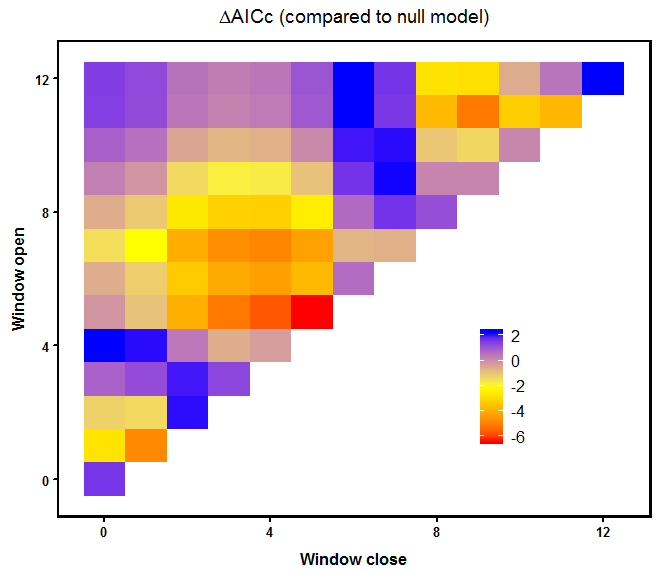 | 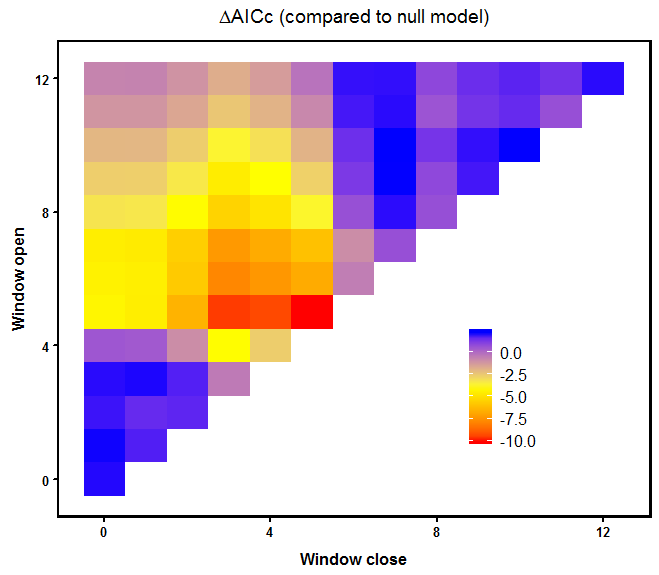 | 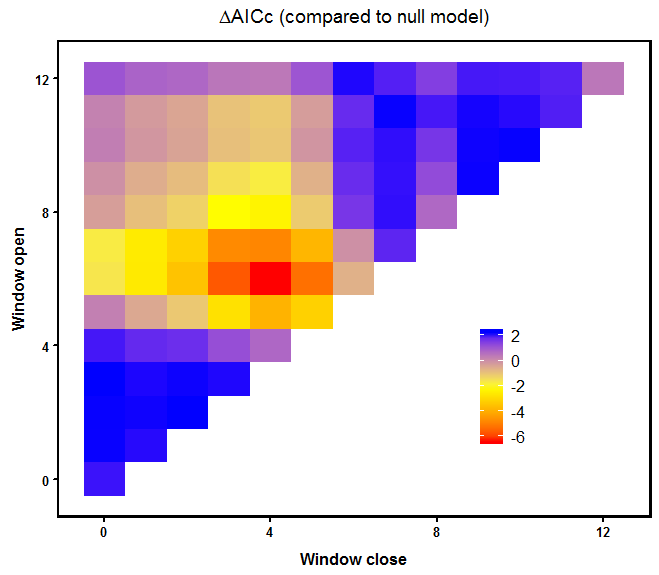 |  |
| Kenema, FL vs. Temperature | Nimba, FL vs. Temperature | Gagnoa, FL vs. Temperature |  |
| 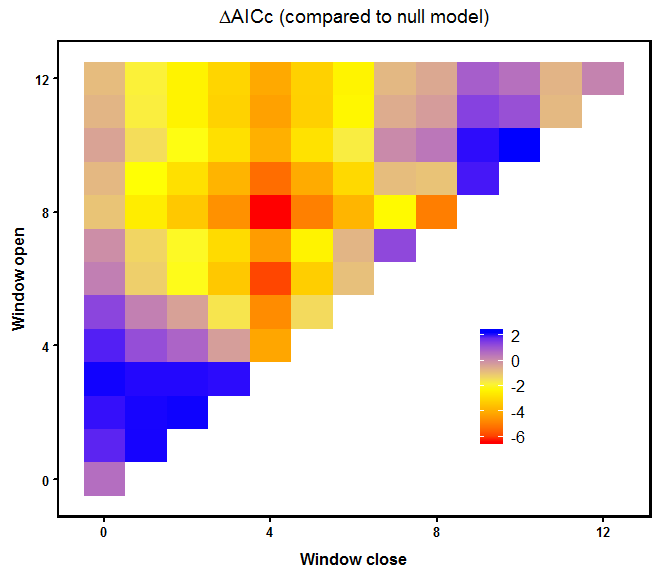 | 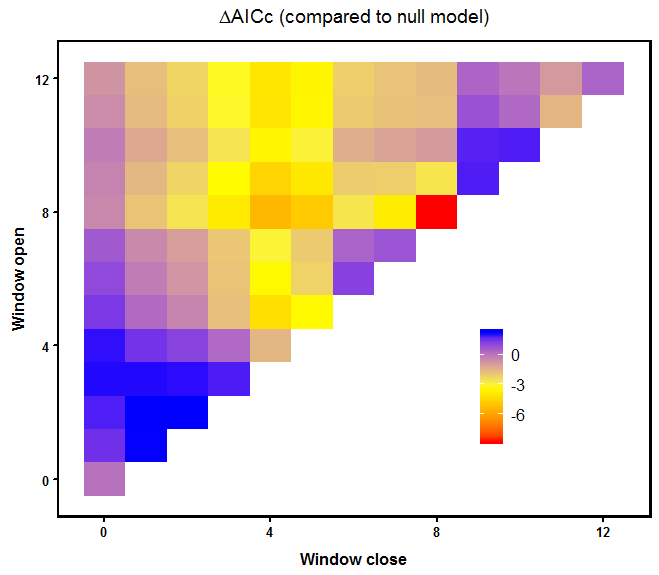 | 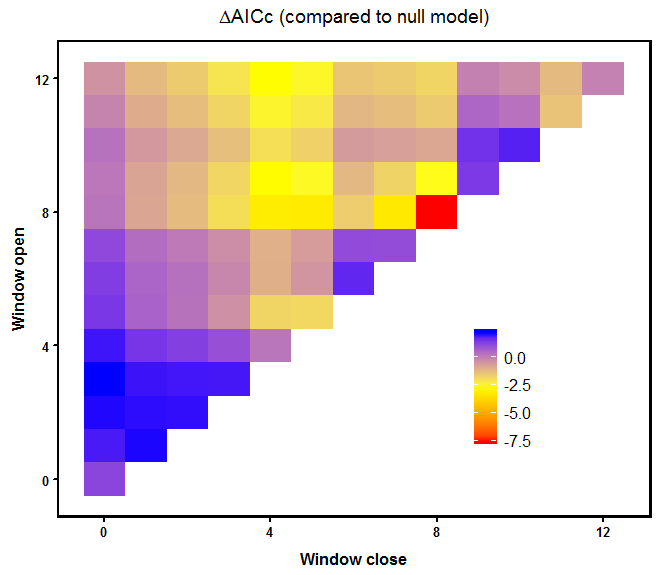 |  |
| Ussel, GR vs. Temperature | Cordoba, GR vs. Temperature | Marrakesh, GR vs. Temperature |  |
| 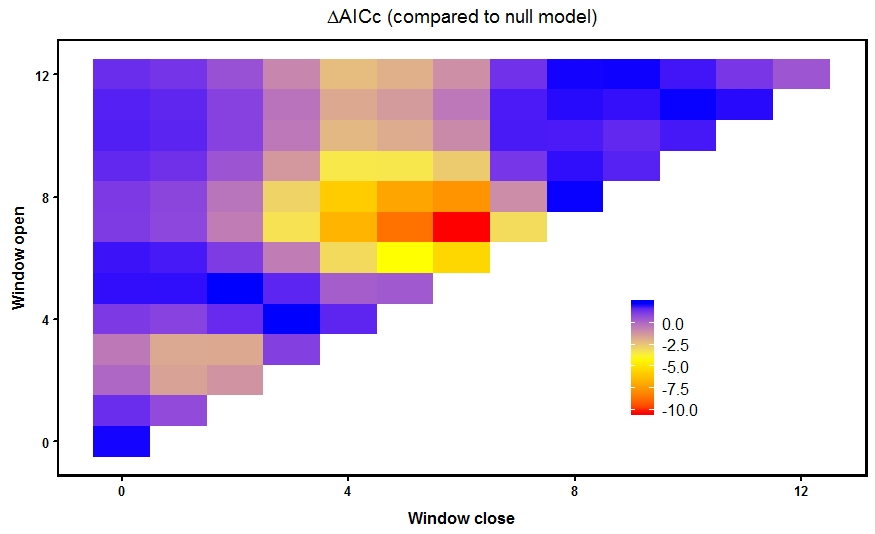 | 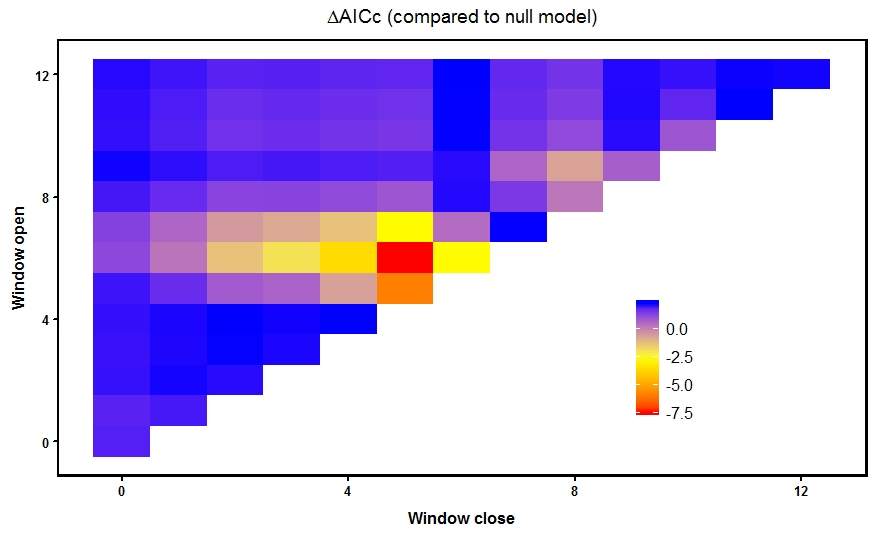 | 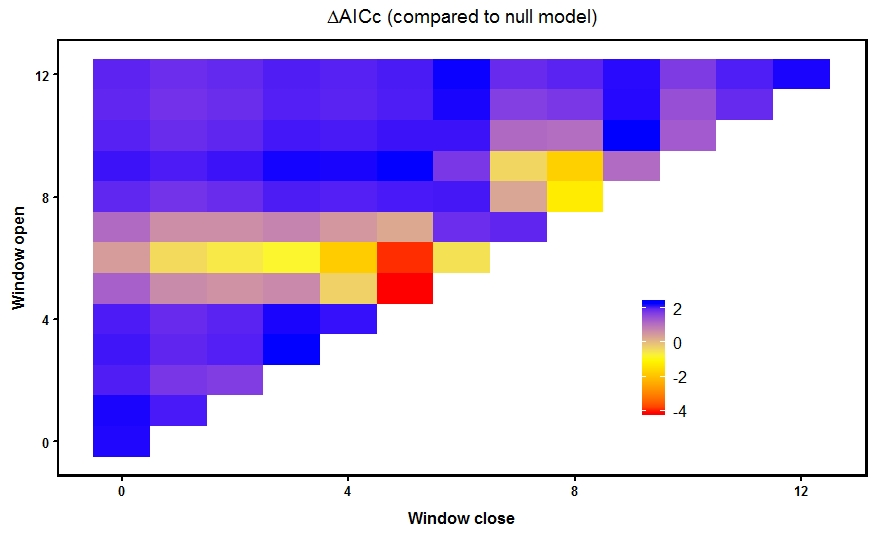 |  |
| Laitila, GR vs. Rainfall | Hamburg, GR vs. Rainfall | Ussel, GR vs. Rainfall |  |
| 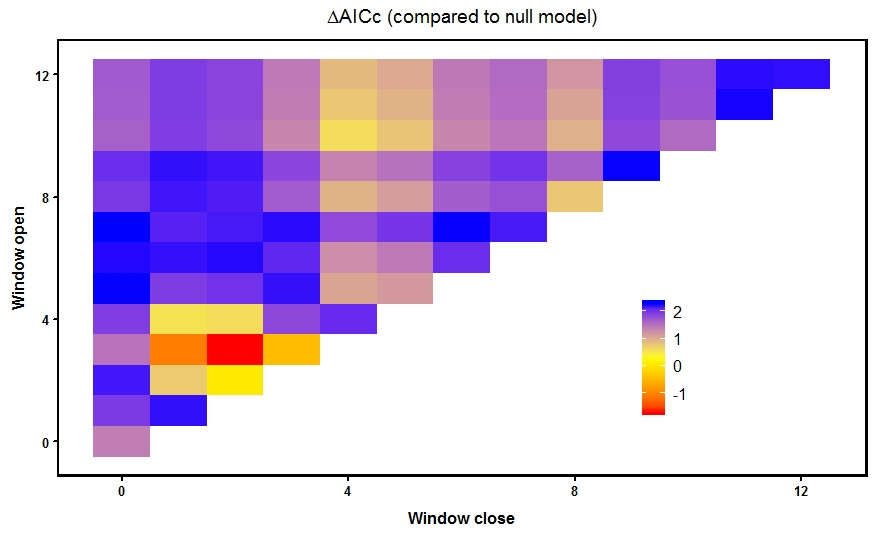 | 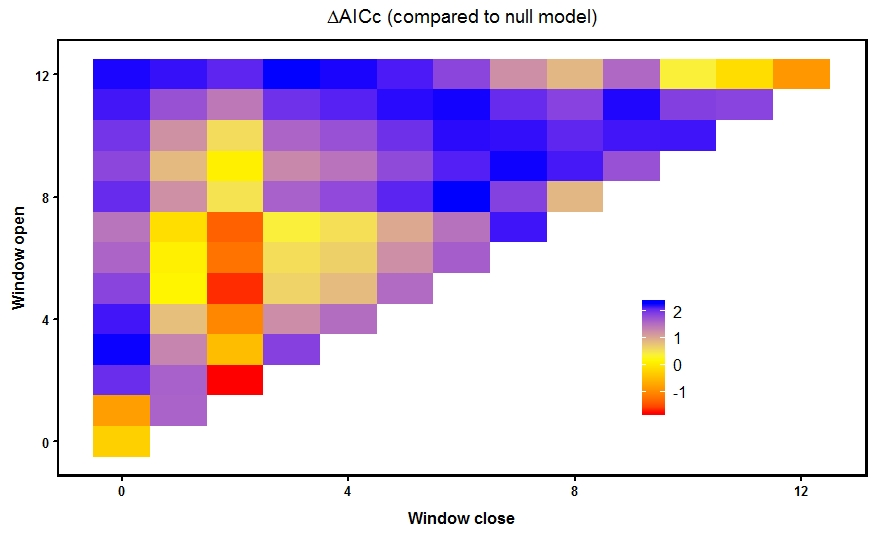 | 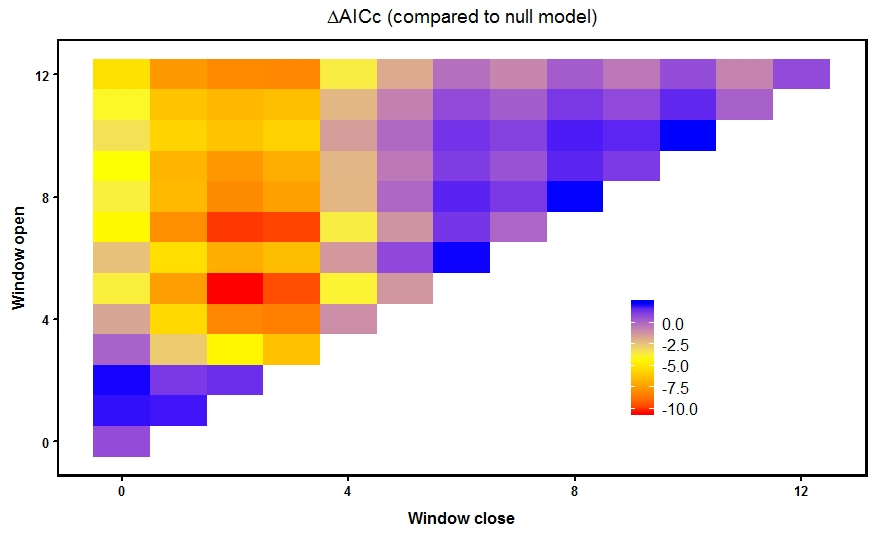 |  |
| Ballou, GR vs. Rainfall | Kenema, GR vs. Rainfall | Nimba, GR vs. Rainfall |  |
| 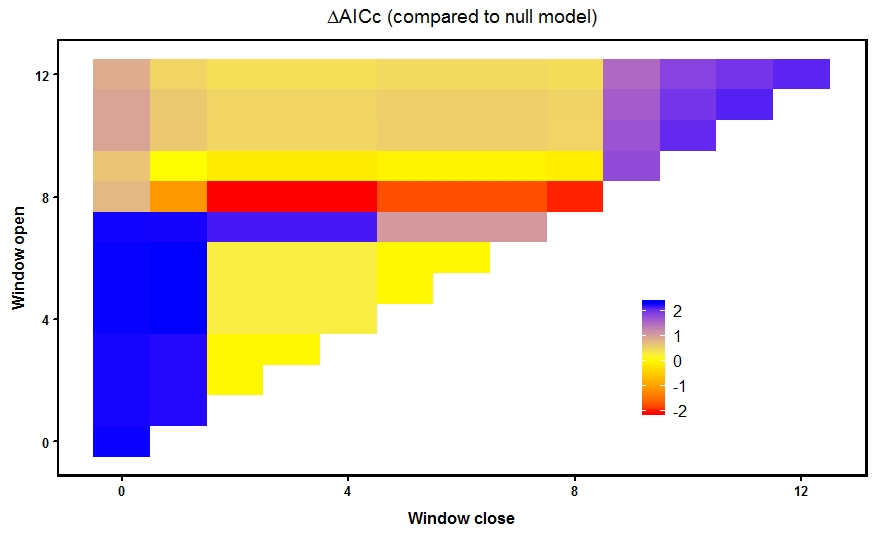 | 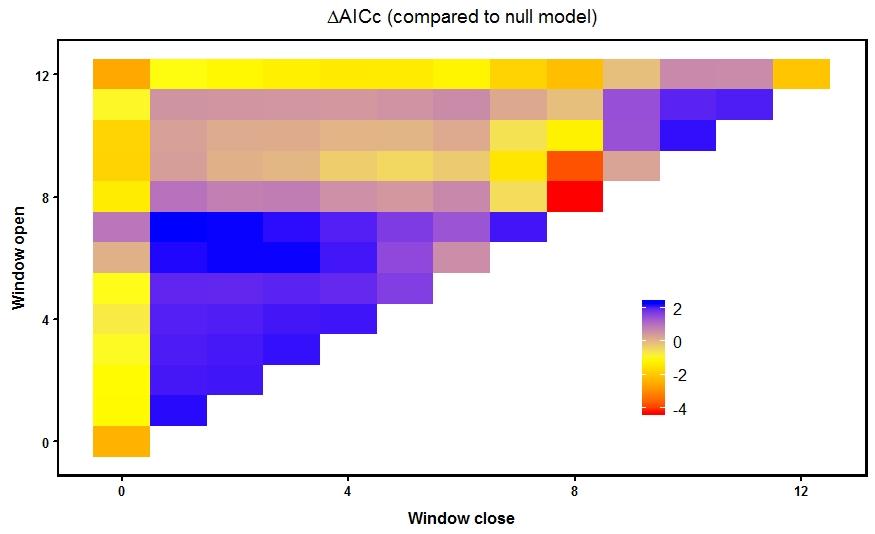 | 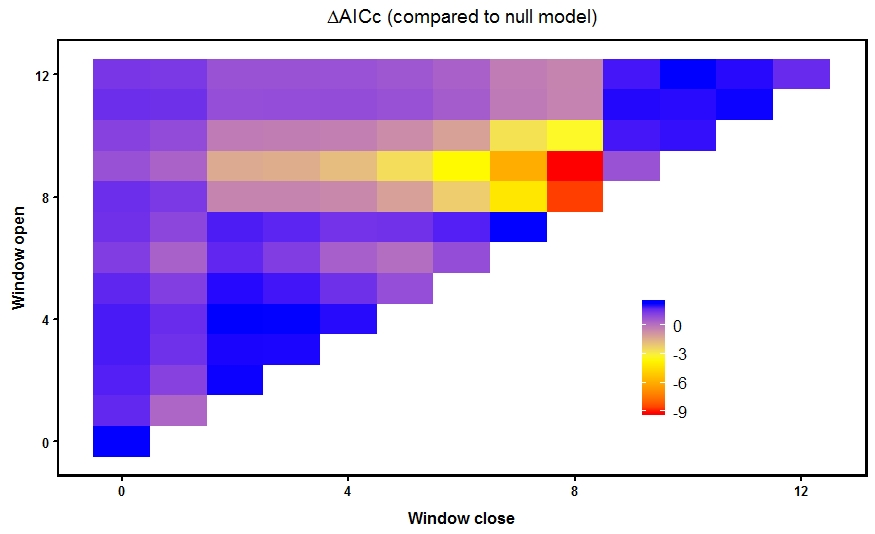 |  |
| **Figure S1**. Delta AICc values from linear models of the Climwin package for the relationships between population parameters (population growth rate [GR] and average annual fledgling number [FL] of the pied flycatcher, *Ficedula hypoleuca*) and two climate variables (monthly mean temperature and rainfall) at breeding, migration and non-breeding grounds. The graphs show all possible models for those locations that were selected to compose climate variables for structural equation models (see Table S1). N = 76 years for fledgling number and 74 years for growth rate. | | |  |

Figure S2. Raw data figures for selected variables used in the SEM models for population growth rate and fledgling number. For fledgling number annual average numbers are presented here, although in the analysis we used nest-specific data.

**Supplement B**

*Details related to dynamic and multilevel structural equation models.*

**Centering of variables in the model for fledgling number**: For variables measured at the within-level (i.e. temperature and precipitation during nestling period, hatching day, clutch size and our outcome, the number of fledglings), the regression slopes associated are also estimated at the between-level as they might influence the response also between the years (van de Pol & Verhulst, 2006). In order to separate the within- and between-level effects of predictors within-cluster centring of the predictors is applied. Usually simple averages of the within-level units are used at the between-level, but this may be problematic as these averages can be contaminated by measurement error due to few within-subject measurements, thus biasing the estimates (Lüdtke et al., 2008; Preacher et al., 2016). For example, for some of the years we have only less than ten nests recorded compared to years with >200 nests. As a solution to this problem, we used the latent mean centring that treats both the within- and between-level components as uncorrelated latent variables to separate within- and between-level effects (Lüdtke et al., 2008; Aspaurohov & Muthén 2019). As such, this approach can be viewed as an implicit latent group-mean centring of the latent within-level variable (Muthén et al., 2016). Interpretation of the regression coefficients in this setting is as follows: at the within-level, the coefficients tell us how much the responses change when the predictors relatively change one unit within a year (i.e. one egg more increases the number of fledglings by 0.7 chicks in nests relatively to that year) whereas at the between-level those coefficients tell how much the responses change on average when the predictors change on average one unit between the years (i.e. if average clutch size across all nests changes one unit, average fledgling number increases by 0.7 chicks). Variables measuring climatic windows (i.e., variables already measured only at the between-level) were grand-mean centred.

**An interaction between within-level temperature and precipitation at the nestling period.** Latent moderated structural equations (LMS) method was applied in order to examine the interaction between nestling period temperature and precipitation at the within-level (Preacher et al. 2016). We were not interested in this interaction at the between-level.

**Bayesian estimation.** Both models were analysed using Bayesian estimation. The benefits of Bayesian methods over traditional frequentist estimation here relate mainly for the model for population growth rate: it has benefits in terms of the ability to estimate more complex models with smaller sample size (Lee & Song, 2004) and better control of Type I error rate and statistical power, including also in mediation modelling framework (Miočević et al. 2017). However, recent research has shown that the better small sample performance of Bayesian approach is achieved only when informative priors (i.e., a plausible range for the possible values are given) are used instead of non-informative ones (McNeish, 2016; Smid et al., 2020). We thus used weakly informative priors for the parameters of main interest in the population growth rate model, an approach that has recently been recommended in the ecological literature as well (Lemoine, 2019). That is, by specifying weakly informative priors we aimed at excluding regression estimates that, based on our knowledge on the study system, would be clearly biologically implausible. Hyperparameters for all the priors of the estimated parameters are given in Table S2. For example, a weakly informative prior for a regression coefficient of previous year’s fledgling number on population growth rate was given as Normal (0,0.1^2^) where a normally-distributed prior has a mean of zero with a standard deviation of 0.1 (Table S2). This specification puts 95% prior probability mass of this regression coefficient in between [-0.196, 0.196]. Since population growth rate is log-transformed, this means that, e.g., a one-unit increase in previous year’s fledgling number is very unlikely to increase population growth rate more than 21.7%. Please note that we applied informative priors for only (non-autoregressive) regression coefficients and for the intercept of population growth rate and density, and not to all parameters involved in RDSEM. The informative priors for the intercept of population growth rate and density were used to improve sampling (i.e., to avoid large spikes of parameter space in trace plots) in comparison to non-informative priors.

As recommended in the case of informative priors (Depaoli & van de Schoot, 2017; van Erp et al., 2018), we ran a prior sensitivity check by repeating the analysis using different prior choices for those regression parameters. As shown in Table S3, our results and biological conclusions seem robust to different prior specifications that still have some biological reality.

Both models were fitted using Gibbs sampler (maximum likelihood estimates were used as starting values for the fledgling number model). Median of posterior distribution was used as a point estimate and the highest posterior density (HPD) was used for interval estimation. Missing data was assumed to be missing at random (MAR), i.e., full-information was used for parameter estimation. Irrespective of some of the responses being count variables (the number of fledglings and clutch size), their distributions were reasonably normally distributed and showed no strong truncation. Therefore, we treated all the responses as continuous variables since this greatly simplifies the calculation of indirect and total effects. Since sample size was not an issue for fledgling number model, non-informative normally-distributed vague priors were used for intercepts and means (whether related to observed or latent variables) and regression coefficients (N(0,100^2^)), non-informative inverse Gamma priors (IG(-1,0)) for variances and residual variances of observed variables and non-informative inverse Wishart priors (IW(0, −3)) for variances and residual variances of latent variables as well as their covariances. Four chains with a total of 100,000 iterations were run (with a burn-in of 50,000 iterations). Convergence of chains was determined using potential scale reduction value, which was 1.001 and 1.000 in nestling and growth rate models after the iterations, respectively, and inspection of individual trace plots of individual parameters (convergence was also verified by doubling the number of iterations). Autocorrelation plots of parameters showed no high autocorrelation at large lags.

Aspaurohov, T. & Muthén, B. (2019) Latent variable centering of predictors and mediators in multilevel and time-series models. *Structural Equation Modeling:* *A multidisciplinary Journal,* 26, 119–142.

Depaoli, S. & van de Schoot, R. (2017) Improving transparency and replication in Bayesian statistics: The WAMBS-checklist. *Psychological Methods*, 22, 240–261.

Lee, S. Y. & Song, X. Y. (2004) Evaluation of the Bayesian and maximum likelihood approaches in analyzing structural equation models with small sample sizes. *Multivariate Behavioral Research,* 39, 653–689.

Lemoine, N. P. (2019) Moving beyond noninformative priors: why and how to choose weakly informative priors in Bayesian analyses. *Oikos*, 128, 912–928.

Lüdtke, O., Marsh, H. W., Robitzsch, A., Trautwein, U., Asparouhov, T. & Muthén, B. (2008) The multilevel latent covariate model: A new, more reliable approach to group-level effects in contextual studies. *Psychological Methods*, 13, 203–229.

McNeish, D. (2016) On using Bayesian methods to address small sample problems. *Structural Equation Modeling: A Multidisciplinary Journal*, 23, 750–773.

# Miočević, M., [MacKinnon](https://www.ncbi.nlm.nih.gov/pubmed/?term=MacKinnon%20DP%5BAuthor%5D&cauthor=true&cauthor_uid=29662296), D. P. & [Levy](https://www.ncbi.nlm.nih.gov/pubmed/?term=Levy%20R%5BAuthor%5D&cauthor=true&cauthor_uid=29662296), R. 2017 Power in bayesian mediation analysis for small sample research. *Structural Equation Modeling: A Multidisciplinary Journal*, 24, 666–683.

Muthén, B., Muthén, L. K. & Aspaurohov, T. (2016) *Regression And Mediation Analysis Using Mplus*. Muthén and Muthén, Los Angeles, CA.

Preacher, K. J., Zyphur, M. J. & Zang, Z. (2010) A general multilevel SEM framework for assessing multilevel mediation. *Psychological Methods*, 15, 209–233.

Preacher, K. J., Zang, Z. & Zyphur, M. J. (2016) Multilevel structural equation models for assessing moderation within and across levels of analysis. *Psychological Methods*, 21, 189–205.

Smid, S. C., McNeish, D., Miočević, M. & van de Schoot, R. (2020) Bayesian versus frequentist estimation for structural equation models in small sample contexts: A systematic review. *Structural Equation Modeling: A Multidisciplinary Journal*, 27, 131–161*.*

van de Pol, M. & Verhulst, S. (2006) Age-dependent traits: a new statistical model to separate within- and between-individual effects. *American Naturalist*, 167, 766–773.

van Erp, S. J., Mulder, J. & Oberski, D. L. (2018) Prior sensitivity analysis in default Bayesian structural equation modeling. *Psychological Methods*, 23, 363–388.

| **Table S2**. Prior specifications for the residual dynamic structural equation model. Please note that population growth rate is measured on the log scale. | | | | | |
| --- | --- | --- | --- | --- | --- |
|  |  |  | Hyperparameter value |  | 95% probability mass |
| Parameter | | | distribution (location, variance) |  | in between |
| *Structural coefficients* | | |  |  |  |
|  | Population growth rate on | |  |  |  |
|  |  | Population density index _t-1_ | Normal (0, 0.1^2^) |  | [-0.196, 0.196] |
|  |  | Number of fledglings _t-1_ | Normal (0.1, 0.1^2^) |  | [-0.096, 0.296] |
|  |  | Number of fledglings _t-2_ | Normal (0, 0.1^2^) |  | [-0.196, 0.196] |
|  |  | Mediterranean winter temperature | Normal (0, 0.1^2^) |  | [-0.196, 0.196] |
|  |  | European spring precipitation | Normal (0, 0.05^2^) |  | [-0.098, 0.098] |
|  |  | African autumn precipitation | Normal (0, 0.01^2^) |  | [-0.0196, 0.0196] |
|  | Population density index on | |  |  |  |
|  |  | Number of fledglings _t-2_ | Normal (15, 15^2^) |  | [-29.4, 39.9] |
|  | Autoregressive coefficients | | Normal (0, ∞) |  | [-∞, ∞] |
|  |  |  |  |  |  |
| *Means* | | |  |  |  |
|  | Number of fledglings _t-1_ | | Normal (0, ∞) |  | [-∞, ∞] |
|  | Number of fledglings _t-2_ | | Normal (0, ∞) |  | [-∞, ∞] |
|  |  |  |  |  |  |
| *Intercepts* | | |  |  |  |
|  | Population growth rate | | Normal (0, 0.3^2^) |  | [-0.58, 0.58] |
|  | Population density index _t-1_ | | Normal (50, 15^2^) |  | [20.60, 79.40] |
|  |  |  |  |  |  |
| *Variance* | | |  |  |  |
|  | Number of fledglings _t-1_ | | Inverse Gamma (-1,0) |  | [-∞, ∞] |
|  | Number of fledglings _t-2_ | | Inverse Gamma (-1,0) |  | [-∞, ∞] |
|  |  |  |  |  |  |
| *Residual variances* | | |  |  |  |
|  | Population growth rate | | Inverse Gamma (-1,0) |  | [-∞, ∞] |
|  | Population density index _t-1_ | | Inverse Gamma (-1,0) |  | [-∞, ∞] |

| **Table S3.** Prior sensitivity analysis of the weakly informative priors used in a model for population growth rate. The first column gives the results for the weakly informative priors used in the main analysis and the second column for non-informative priors. In the subsequent columns, the prior mean and variance of these parameters are altered and the models rerun (prior variance was reduced only since allowing infinite variance (i.e. non-informative priors) for the parameter return almost identical estimates compared to weakly informative priors). These results suggest that these alterations have very small influences on the parameter estimates and, thus, on the biological conclusions drawn here. For example, in the 3rd and 5th columns prior means are changed by 0.2 units which in most cases corresponds to what we would consider as biologically unrealistic values in this context. | | | | | | | | | | |
| --- | --- | --- | --- | --- | --- | --- | --- | --- | --- | --- |
|  |  | Weakly informative priors | Non-informative Mplus | Increment of | Increment of | Decrease of | Decrease of | Decrease of | Decrease of | Decrease of |
|  |  | (from Table S2) | defaults (N(0, ∞)) | .2 to the prior mean | .5 to the prior mean | .2 to the prior mean | .5 to the prior mean | 20% of prior variance | 50% of prior variance | 80% of variance |
| Annual population growth rate | |  |  |  |  |  |  |  |  |  |
|  | Population density _t-1_ | -0.003  (-0.005, -0.0012) | -0.003  -0.005, -0.002) | -0.003  -0.005, -0.002) | -0.003  -0.005, -0.002) | -0.003  (-0.005, -0.002) | -0.003  (-0.005, -0.002) | -0.003  (-0.005, -0.002) | -0.003  -0.005, -0.002) | -0.003  (-0.005, -0.002) |
|  | Number of fledglings _t-1_ | 0.028  (0.005, 0.051) | 0.027  0.004, 0.051) | 0.031  0.008, 0.055) | 0.036  0.012, 0.057) | 0.025  0.002, 0.049) | 0.021  -0.002, 0.044) | 0.028  0.005, 0.052) | 0.029  0.006, 0.052) | 0.032  (0.009, 0.055) |
|  | Number of fledglings _t-2_ | -0.004  (-0.026, 0.018) | -0.004  -0.026, 0.018) | -0.002  -0.024, 0.020) | 0.002  -0.020, 0.025) | -0.007  -0.029, 0.015) | -0.011  -0.035, 0.011) | -0.004  -0.026, 0.017) | -0.004  -0.026, 0.017) | -0.005 (-0.026, 0.017) |
|  | Mediterranean winter temperature | -0.025  (-0.045, -0.006) | -0.026  -0.045, -0.006) | -0.023  -0.043, -0.004) | -0.020  -0.039, 0.001) | -0.027  -0.047, -0.008) | -0.031  -0.051, -0.011) | -0.025  -0.044, -0.006) | -0.025  -0.044, -0.006) | -0.024  -0.043, -0.005) |
|  | European spring precipitation | -0.00166  (-0.00354, 0.00025) | -0.00169  -0.00359, 0.00021) | -0.00158  -0.00347, 0.00036) | -0.00144  -0.00340, 0.00057) | -0.00173  -0.00362, 0.00018) | -0.00185  -0.00379, 0.00008) | -0.00165  -0.00355, 0.00025) | -0.00163  -0.00351, 0.00029) | -0.00155  -0.00343, 0.00036) |
|  | African autumn precipitation | -0.00066  (-0.00120, -0.00013) | -0.00066  -0.00120, -0.00014) | -0.00051  -0.00104, 0.00004) | -0.00026  -0.00082, 0.00032) | -0.00018  -0.00134, -0.00027) | -0.00105  -0.00163, -0.00050) | -0.00066  -0.00119, -0.00013) | -0.00066  -0.00191, -0.00013) | 0.00065  -0.00119, -0.00012) |
| Population density | |  |  | N  20, 225) | N  25, 225) | N  15, 225) | N  5, 225) |  |  |  |
|  | Number of fledglings _t-2_ | 4.34  1.82, 6.90) | 4.26  1.71, 6.81) | 4.37  1.84, 6.93) | 4.39  1.87, 6.96) | 4.35  1.80, 6.87) | 4.31  1.75, 6.82) | 4.36  1.84, 6.92) | 4.42  1.91, 6.96) | 4.65  2.16, 7.15) |

Supplement C

Excel file for the data used in the population growth analysis
